# Soil Biological Activity Contributing to Phosphorus Availability in Vertisols under Long-Term Organic and Conventional Agricultural Management

**DOI:** 10.3389/fpls.2017.01523

**Published:** 2017-09-04

**Authors:** Nisar A. Bhat, Amritbir Riar, Aketi Ramesh, Sanjeeda Iqbal, Mahaveer P. Sharma, Sanjay K. Sharma, Gurbir S. Bhullar

**Affiliations:** ^1^Government Holkar Science College, Devi Ahilya Vishwavidyalaya Indore, India; ^2^Department of International Cooperation, Research Institute of Organic Agriculture (FiBL) Frick, Switzerland; ^3^ICAR-Indian Institute of Soybean Research Indore, India; ^4^Rajmata Vijayaraje Scindia Krishi Vishwavidyalaya Agriculture College Indore, India

**Keywords:** biological properties, phosphorus mobilization, soil enzymes, soybean–wheat system, available P

## Abstract

Mobilization of unavailable phosphorus (P) to plant available P is a prerequisite to sustain crop productivity. Although most of the agricultural soils have sufficient amounts of phosphorus, low availability of native soil P remains a key limiting factor to increasing crop productivity. Solubilization and mineralization of applied and native P to plant available form is mediated through a number of biological and biochemical processes that are strongly influenced by soil carbon/organic matter, besides other biotic and abiotic factors. Soils rich in organic matter are expected to have higher P availability potentially due to higher biological activity. In conventional agricultural systems mineral fertilizers are used to supply P for plant growth, whereas organic systems largely rely on inputs of organic origin. The soils under organic management are supposed to be biologically more active and thus possess a higher capability to mobilize native or applied P. In this study we compared biological activity in soil of a long-term farming systems comparison field trial in vertisols under a subtropical (semi-arid) environment. Soil samples were collected from plots under 7 years of organic and conventional management at five different time points in soybean (*Glycine max*) -wheat (*Triticum aestivum*) crop sequence including the crop growth stages of reproductive significance. Upon analysis of various soil biological properties such as dehydrogenase, β-glucosidase, acid and alkaline phosphatase activities, microbial respiration, substrate induced respiration, soil microbial biomass carbon, organically managed soils were found to be biologically more active particularly at R2 stage in soybean and panicle initiation stage in wheat. We also determined the synergies between these biological parameters by using the methodology of principle component analysis. At all sampling points, P availability in organic and conventional systems was comparable. Our findings clearly indicate that owing to higher biological activity, organic systems possess equal capabilities of supplying P for crop growth as are conventional systems with inputs of mineral P fertilizers.

## Introduction

Low availability of native soil phosphorus for plant growth acts as a limiting factor to realize increased crop productivity (Lynch and Brown, 2008; Khan and Joergensen, 2009; Malik et al., 2012; Johnston et al., 2014). It is well known that most of the soils contain appreciable amounts of total P, yet soil solution P concentrations are ironically low and thereby an impediment for sufficient plant P assimilation (Hinsinger, 2001). As P is subjected to precipitation reactions and sorption reactions on soil colloids, substantial proportions of applied and native soil P are rendered unavailable (Alam and Ladha, 2004; Brady and Weil, 2008; Khan and Joergensen, 2009). Therefore, owing to the very low efficiency of applied P (Syers et al., 2008), large amounts of fertilizer P are required to sufficiently increase soil solution P concentrations for assimilation by crop plants to sustain crop productivity (Zhang et al., 2010; Shen et al., 2011; Bai et al., 2013). Inorganic P fertilizers are, however, costly and are either out of the reach of resource poor farmers in most of the developing countries or need to be heavily subsidized by tax payers’ money. Furthermore, with rapidly diminishing accessible natural P resources, relying solely on inorganic P fertilizers is not a sustainable strategy (Cordell et al., 2009). Therefore, it is of high importance that alternate agricultural management strategies are devised that are cost effective, P efficient and sustainable (Harvey et al., 2009; Sánchez, 2010). Apart from the input of mineral P fertilizers, some of the agricultural strategies that can mobilize soil P for plant assimilation include organic matter management (Damodar Reddy et al., 1999; Aulakh et al., 2003; Singh et al., 2007), tillage interventions (Basamba et al., 2006; Shi et al., 2013), microbial inoculation (Ramesh et al., 2011, 2014; Kumar et al., 2014), and crop rotation (Aulakh et al., 2003; Ciampitti et al., 2011).

In nature, phosphorus is known to occur in a number of discrete chemical forms varying in solubility and availability. In agricultural soils, P is found in both inorganic and organic forms, of which organic forms of P are predominant (Turner et al., 2002; Condron et al., 2005; Kong et al., 2009; Richardson et al., 2011). Most of the organic P exists as phytate-P and in lesser amounts as other phosphate esters such as phospholipids (Turner et al., 2007; Richardson et al., 2011). The presence of high phytate-P in soils could be attributed to its low solubility and close affinity toward the solid phase (soil colloids) because of its higher stability (George et al., 2005; Tang et al., 2006). This has been a major impediment to P availability for plant uptake. Availability of P for crop assimilation is net resultant of a number of simultaneously occurring processes, predominantly the mobilization of inorganic P, mineralization of organic P, immobilization of applied P and the rates of P diffusion. These processes are influenced and mediated by several bio-chemical and microbiological activities. Though the roles of most of these biological activities in specific processes are well understood, their synergistic or antagonistic functions and their interactions under particular management environments are still poorly studied.

By improving soil physicochemical and biological properties, organic farming systems are known to play an important role in agricultural ecosystems. They are also advocated for their contribution to nutrient cycling in general and P in particular (Malik et al., 2013; Masto et al., 2013; Tamilselvi et al., 2015). Organic matter contributes 20–80% to the organic phosphorous in soil (Richardson, 1994), which in turn is hydrolyzed by phosphatases – enzymes of plant or microbial origin – to become plant available P (Tarafdar and Claassen, 1988). Not only does the mineralization of organic manure supplies available P for plant uptake, it also plays a significant role in mobilization of native P forms through an array of mechanisms. For instance, organic anions evolved during manure decomposition, metal complexation or dissolution reactions mediate release of P from exchange sites (Bolan et al., 1994; Iyamuremye and Dick, 1996). Also, the addition of organic matter serves as a substrate for microbial proliferation that aides in changing the dynamics of P (both organic and inorganic forms) in the rhizosphere thereby positively affecting root architecture and biological properties such as root released phosphatases or phosphatases of microbial origin or both (Gichangi et al., 2009; Richardson et al., 2011; Guan et al., 2012; Malik et al., 2013). The effectiveness of added organic manures on microbial activity can be ascertained by assessing its influence on pertinent changes in soil properties such as pH, soil enzymatic activities, microbial biomass and its role in P mobilization and assimilation. The assay of soil enzymatic activities could provide an early and sensitive indication of changes induced by management strategies such as organic manuring, green manuring, crop residue incorporation, tillage interventions, herbicide application etc. (Dick et al., 1988; Nannipieri, 1994; Aparna et al., 2014; Tamilselvi et al., 2015). Enzyme activity coupled with measurements of other relevant biological and biochemical parameters (e.g., soil respiration, microbial carbon biomass, soil pH etc.) provides indication on the extent of biological activity in soil. Because of the inherent complexity of multiple co-existing soil processes, it is, however, challenging to quantify the net contribution of each of these factors to plant P-availability under specific production systems.

The proclaimed effectiveness of organic management in enhancing P availability could only be determined by systematic comparison with conventional management systems under field conditions. Such comparative studies need to also consider the minimum time required for organic systems to become fully functional. Despite the fact that P availability in soils is of high scientific interest, systematic long-term comparisons of factors contributing to P availability in organic and conventional farming systems are lacking. In this study, we compared soil biological activities pertaining to P availability at key crop growth stages in agricultural plots that were subject to continuous organic and conventional management for 7 years. The study was conducted within the framework of a long-term farming systems comparison trial in Vertisols of Central India, where soybean (*Glycine max*) – wheat (*Triticum aestivum*) is a predominant cropping system. We hypothesized that biological activity in soils of organic production systems plays a significant role in P mobilization in a soybean–wheat cropping system. The specific objective of this study was to monitor changes in and synergies among soil biological parameters contributing to P availability such as soil dehydrogenase activity (DHA), β-glucosidase (βGL), acid phosphatase (ACP) and alkaline phosphatase (ALP) activities, soil microbial respiration (SR), substrate induced respiration (SIR) and soil microbial biomass carbon (MBC) content at key growth stages of soybean and wheat crops.

## Materials and Methods

### Site and Trial Description

**FIGURE 1:**
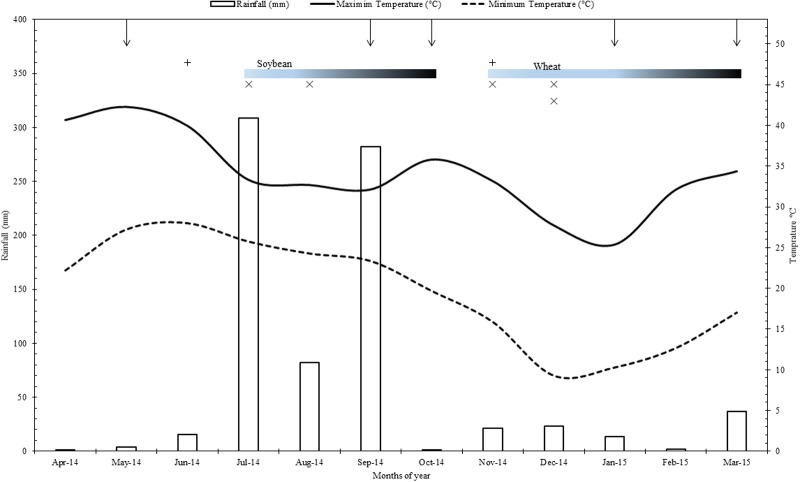
Rainfall, minimum and maximum temperatures of the experimental site during the period of study (2014–2015). Where, vertical arrows from top horizontal axis represent the time of sampling and gradient bars indicate the crop growth periods for Soybean and wheat; +, Farmyard + rock phosphate and compost application in organic systems; x, fertilizers application in conventional systems.

This study was conducted on the field site of the long term farming systems comparison (SysCom) trial running since 2007 in the Nimar valley of Madhya Pradesh state in central India. The trial site is located at an altitude of 250 m above sea level (22°8′30.28″N; 75°37′48.97″E) in a subtropical (semi-arid) climate with an average temperature of 25°C (temperature range 05–48°C) of which the maximum temperature occurs during May/June and minimum temperature during January/February. This region receives an average precipitation of 800 mm, most of which comes during monsoon period from June to September (**Figure 1**). The experimental site belongs to Vertisols (Fine, iso-hyperthermic, montmorillonitic, Typic Haplusterts) and the pertinent soil characteristics at the start of the experiment in 2007 were pH 8.7, organic carbon content 5.0 gkg^-1^, clay content 600 gkg^-1^, CaCO_3_ 55 gkg^-1^, and available (Olsen’s) P content of 7.0 mg kg^-1^ (Forster et al., 2013). Cotton/ soybean–wheat is the predominant cropping pattern in Nimar valley, though farmers also grow other crops such as sugarcane, vegetables, fodder, and pulses.

As described by Forster et al. (2013), the field site of SysCom trial was under conventional management until December 2006, when a test crop of unfertilized wheat was grown to assess the homogeneity of the terrain before setup of the trial. The trial consists of four treatments – two organic farming system, i.e., organic (BIOORG) and biodynamic (BIODYN) and two conventional farming systems, i.e., conventional (CON) and conventional including Bt-cotton (CONBtC). These management systems are replicated four times in a randomized block design in two stripes of plots with gross plot size of 16 m × 16 m and net plot size of 12 m × 12 m. While designing the treatment compositions, due consideration was given to prevalent practices of local farmers as well as standard recommendations. As a rule of thumb, organic management systems are implemented according to the standards prescribed by International Federation of Organic Agriculture movements (IFOAM, 2006) and conventional management is carried out in line with the recommendations of Indian Council of Agricultural Research, with slight adaptations to suit the prevailing local situations (Forster et al., 2013). The nutrient inputs in organically managed plots are mainly supplied by compost, castor cake, rock phosphate, and farm yard manure (FYM), while in conventional management systems, inorganic fertilizers are applied in the form of urea, diammonium phosphate (DAP), Single super phosphate (SSP) and muriate of Potash. It is noteworthy that following the principle of good agricultural practices (and practice of local farmers) every alternative year conventional plots also receive a basal application of 4 t ha^-1^ FYM. This dose of FYM was applied in the previous year (2013). In 2014, conventional system (Soybean + wheat) received a total of 178 kg N ha^-1^, 78 kg P ha^-1^, and 88 kg K ha^-1^ from synthetic mineral fertilizers; whereas organic system received a total of 151 kg N ha^-1^, 79 kg P ha^-1^, and 173 kg K ha^-1^from organic inputs. Soybean crop (variety JS 93-05; seed rate 80 kg ha^-1^) was applied with a basal application of 45 kg P ha^-1^ and 52.5 kg K ha^-1^ from SSP and MOP, respectively, in conventional system. Single dose of 28.5 kg N ha^-1^ from urea was applied at 19 DAS. In organic system, 2.5 t ha^-1^ of FYM and 2.4 t ha^-1^ of acidulated rock phosphate was applied and incorporated in soil by bullock drawn harrow at 34 days before sowing of soybean. Application of FYM and acidulated rock phosphate provided 47 kg N ha^-1^, 33 kg P ha^-1^, and 47 kg K ha^-1^ to wheat crop in organic system. In wheat [variety HI-1544 (Purna), seed rate 100 kg ha^-1^], after soybean, basal application of 33 kg P ha^-1^ and 35 kg K ha^-1^ was applied with SSP and MOP, respectively, in conventional. A total of 149 kg of N ha^-1^ was applied in two identical splits at 19 and 43 DAS. Three days before sowing 13 t ha^-1^ of compost was applied to organic system and incorporated in soil by bullock drawn harrow, which provided 105 kg N ha^-1^, 47 kg P ha^-1^, and 126 kg K ha^-1^. All the cultural management practices such as weed and pest management were followed as per standard norms prescribed for organic and conventional systems (Forster et al., 2013).

### Soil Sampling and Analysis

Organic (BIOORG) and conventional (CON) system plots were sampled during soybean and wheat crops at five different time points, i.e., from fallow land before sowing of soybean, R2 stage of soybean, before sowing of wheat, panicle initiation stage of wheat and after harvest of wheat (**Figure 1**). R2 stage of soybean and panicle initiation stage of wheat are of high reproductive significance and thus important for crop productivity. Each plot was sampled to 0–20 cm depth from six random locations and collected samples were pooled for analysis. DHA was assessed through the reduction of 2,3,5- triphenyltetrazolium chloride (TTC) to triphenylformazan (TPF) using colorimetric procedure (Shimadzu UV-VS, Model- 1800) of Tabatabai (1994) and expressed as μg triphenylformazan g^-1^ soil h^-1^ (Klein et al., 1971). βGL activity was determined using *p*-nitrophenyl-β-D-glucopyranoside (PNG, 0.05M) as a substrate (Sinsabaugh et al., 1999) and the amount of *p*-nitrophenol released was determined spectrophotometrically at OD_420_ and expressed as μg *p*-nitrophenol g^-1^ soil h^-1^ (Tabatabai, 1994). ACP and ALP were assayed by the standard method of Tabatabai and Bremner (1969) in acetate buffer (pH 5.4) and borax-NaOH buffer (pH 9.4), respectively, using *p*-nitrophenyl phosphate as a substrate. Soil pH was determined in a soil: water ratio of 1:2.5 with intermittent stirring for 30 min and feeding directly to a pH meter (Baruah and Barthakur, 1999). SR was determined by quantifying the carbon dioxide released in the process of microbial respiration during 10 days of incubation (Anderson and Domsch, 1990). SIR was determined by quantifying the carbon dioxide released in the process of microbial respiration during 2 h incubation after adding (0.0625 g) glucose and (2.5 g) talc to soil (Anderson and Domsch, 1978). Microbial biomass-Carbon was estimated by employing the fumigation-extraction procedure of Vance et al. (1987) and was calculated from the relationship *Bc* = *Fc/Kc*, where *Fc* is the difference between extractable carbon from fumigated soil and non-fumigated soil; *Kc* is conversion factor, which is 0.45 and the value has been expressed in mg C kg^-1^ soil (Joergensen and Mueller, 1996). Olsen P was extracted with 0.5 M sodium bicarbonate (pH 8.5) in 1:5 ratio of soil to extractant and shaken for 30 min at 150 rpm (Olsen et al., 1954). After filtration of suspension, phosphorus concentration in the extract was estimated colorimetrically by ascorbic acid reductant method (Watanabe and Olsen, 1965). For P content of seed and straw, samples collected from soybean and wheat crops were air-dried and kept in an oven at 65°C till constant weight. Upon grinding the samples were passed through 0.5 mm sieve and digested in acid mixture of HNO_3_:HClO_4_, 5:4 ratio. The phosphorus concentration in the digest was determined colorimetrically using vanadomolybdate yellow color method. The seed and straw yield of each net plot was recorded and converted to kg ha^-1^.

### Statistical Analysis

The data was analyzed by using SAS statistical software (ver.9.2; SAS Institute., Cary, NC, United States). For microbiological parameters and available P content, three way analysis was carried out involving treatments (Organic, conventional) crops (Soybean, wheat) and periods of sampling and their interactions as fixed factors. The significant differences between means were identified using Fisher least significant differences (LSD) and Tukeys multiple comparison tests at *P* = 0.05. For crop yield and uptake parameters, one way analysis of variance (ANOVA) was carried out using the ANOVA procedure in SAS enterprise guide 4.2 and means separated with LSD and Tukeys multiple comparison tests. In order to obtain a comprehensive picture of potential synergistic interactions among the observed biological and microbiological parameters, a Principle Component Analysis (PCA) was carried out. Principle components thus constructed allowed to define which original variables are responsible for the mean difference between systems. PCA was performed using JMP (SAS Institute Inc.) (Goupy and Creighton, 2007).

## Results

First objective of this study was to monitor changes in soil biological properties pertaining to P cycling in organic and conventional management systems. The assessed soil microbiological and chemical parameters showed considerable variation across systems and crop growth stages. Soil DHA did significantly vary between organic and conventional systems at sowing under soybean cropping. Significant increase of up to 16.3% (66.3 μg triphenylformazon g^-1^ soil 24 h^-1^) and 8.7% (58.7 μg triphenylformazon g^-1^ soil 24 h^-1^) was observed in organic and conventional systems, respectively, at R2 stage as compared to sowing (**Table 1**). At R2 stage, organic management registered 12.9% increase in DHA over conventional system. At harvest, there was a significant decline in DHA in both the agricultural systems as compared to its activity at R2 stage and also it showed significant variation between the agricultural management systems with higher DHA in organically managed systems. In wheat crop, DHA was significantly higher by 49% (100.6 μg triphenylformazon g^-1^ soil 24 h^-1^) in organic management as compared to the conventional system (71.2 μg triphenylformazon g^-1^ soil 24 h^-1^). DHA was relatively higher at active crop growth stages in both soybean and wheat while it decreased at harvest. Organically managed system exhibited higher DHA activity throughout the experimental period, which was on an average 17.2% higher than conventional system (**Table 1**).

**Table 1:** Effect of organic and conventional agricultural management practices on soil dehydrogenase, β-glucosidase, acid phosphatase, and alkaline phosphatase activities at different periods of sampling in soybean and wheat crop.

	Dehydrogenase (μg triphenylformazon g^-1^soil 24 h^-1^)		β-Glucosidase (μg *p*-nitrophenol g^-1^ soil h^-1^)		Acid phosphatase (μg *p*-nitrophenol g^-1^ soil h^-1^)		Alkaline phosphatase (μg *p*-nitrophenol g^-1^ soil h^-1^)	
				
Treatments (T)	Organic	Conventional	Organic	Conventional	Organic	Conventional	Organic	Conventional
Treatment mean	66.9 ± 3.5^a^	57.1 ± 2.8^b^	216.1 ± 6.5^a^	188.7 ± 11.9^a^	198.6 ± 14.5^a^	180.4 ± 14.5^a^	413.4 ± 11.9^a^	332.6 ± 22.8^b^
**Period of sampling (PS)**								
**Soybean**								
At sowing	57.0 ± 1.7^aB^	54.0 ± 4.0^aB^	141.3 ± 5.6^aB^	132.3 ± 9.0^aB^	182.9 ± 15.0^aA^	164.6 ± 12.4^aA^	400.7 ± 23.6^aA^	360.8 ± 13.5^bA^
50 DAS	66.3 ± 3.1^aA^	58.7 ± 2.6^bA^	215.4 ± 4.6^aA^	186.8 ± 4.5^bA^	245.7 ± 5.9^aB^	225.4 ± 8.0^bB^	500.7 ± 18.6^aA^	459.8 ± 29.2^aB^
Harvest	56.6 ± 4.8^aB^	52.0 ± 2.5^aB^	155.9 ± 6.1^aB^	123.4 ± 7.6^aB^	226.1 ± 21.6^aB^	214.1 ± 19.9^aB^	465.8 ± 7.2^aA^	374.7 ± 25.6^bA^
Mean of soybean	60.0^a^	54.2^b^	170.9^a^	147.5^a^	218.2^a^	201.4^a^	455.7^a^	398.4^b^
**Wheat**								
60 DAS	100.6 ± 5.3^aA^	71.2 ± 3.5^bA^	337.8 ± 5.6^aA^	292.1 ± 11.0^bA^	184.5 ± 16.5^aa^	175.2 ± 17.5^aA^	412.3 ± 0.0^aB^	318.9 ± 15.9^bA^
Harvest	54.2 ± 2.5^aB^	49.3 ± 1.2^bB^	230.1 ± 10.5^aB^	208.9 ± 27.3^aB^	154.0 ± 13.7^aA^	123.0 ± 14.9^bC^	287.6 ± 10.2^aC^	148.9 ± 30.0^bC^
Mean of wheat	77.4^a^	60.25^b^	283.95^a^	250.5^a^	169.5^a^	149.1^a^	349.95^a^	233.9^b^


*Data are mean values of four replicates; values followed by the same small letters are not significantly different among organic and conventional practices at *P* = 0.05 (horizontal comparison). Similarly values followed by the same capital letters are not significantly different between different periods of sampling (vertical comparison). Values under ANOVA are probabilities (*P*-values) of the source of variations.*

Significantly higher βGL activity under organic management system was observed at R2 stage of soybean with a difference of 15.3% (215.4 μg *p*-nitrophenol g^-1^ soil h^-1^) to conventional management system (**Table 1**). From sowing to R2 stage of soybean, βGL activity saw a significant increase of 52.4% in organic (215.4 μg *p*-nitrophenol g^-1^ soil h^-1^) and 41.2% (186.8 μg *p*-nitrophenol g^-1^ soil h^-1^) in conventional system and there was a significant decline at harvest. At panicle initiation stage of wheat, βGL activity was 15.7% (337.8 μg *p*-nitrophenol g^-1^ soil h^-1^) higher in organic management system than conventional management system.

Acid phosphatase activity was significant at R2 stage of soybean and at harvest of wheat. In contrast, ALP activity was significantly higher in organic systems as compared to conventional at all the sampling times except for R2 stage of soybean (**Table 1**). Considering the overall average of the soybean–wheat system 24% higher (413.4 μg *p*-nitrophenol g^-1^ soil h^-1^) ALP activity was recorded under organic management compared to the conventional management. MBC increased during the active crop growth stages and decreased toward harvest of soybean and wheat. At each of the sampling points, MBC tended to be higher under organic management than conventional, but the differences were never significant (**Table 2**). Similar was the case of SIR, which increased from second sampling (R2 stage) onward and decreased at harvest of wheat. Organic system exhibited slightly higher MBC than conventional system throughout the experiment but did not attain the level of significance (**Table 2**). SR was higher in organically managed soil before sowing of soybean, at R2 stage and at harvest of soybean. Whereas, in case of wheat, organic and conventional systems were statistically not different for SR at both the sampling points (**Table 2**). Within each management system, SR did not exhibit a major change during the different sampling points except for a significant decline at the harvest of wheat.

**Table 2:** Effect of organic and conventional agricultural management practices on microbial biomass Carbon, substrate induced respiration, microbial respiration, pH, and available phosphorous at different periods of sampling in soybean and wheat crop.

	Microbial biomass carbon (mg C Kg^-1^ soil)		Substrate induced respiration (mg CO_2_ Kg^-1^ h^-1^)		Microbial respiration (mg CO_2_ Kg^-1^ day^-1^)		pH		Available phosphorous (μg g^-1^ soil)	
					
Treatments (T)	Organic	Conventional	Organic	Conventional	Organic	Conventional	Organic	Conventional	Organic	Conventional
Treatment mean	311.7 ± 22.8^a^	294.2 ± 20.2^a^	19.1 ± 1.4^a^	17.5 ± 1.3^a^	16.5 ± 0.5^a^	15.2 ± 0.6^b^	8.02 ± 0.1^a^	7.97 ± 0.2^a^	5.9 ± 0.1^a^	5.6 ± 0.1^b^
**Period of sampling (PS)**									
**Soybean**										
At sowing	270.8 ± 25.0^aA^	250.0 ± 13.6^aA^	13.8 ± 2.3^aA^	12.6 ± 1.2^aA^	16.3 ± 0.7^aA^	14.6 ± 0.5^bA^	8.09 ± 0.0^aA^	8.05 ± 0.1^aA^	5.4 ± 0.1^aA^	5.2 ± 0.2^aA^
50 DAS	391.7 ± 21.5^aB^	362.5 ± 25.0^aB^	22.2 ± 1.2^aB^	20.4 ± 1.4^aB^	17.4 ± 0.5^aA^	15.2 ± 0.9^bA^	8.07 ± 0.1^aA^	8.08 ± 0.2^aA^	7.6 ± 0.1^aB^	7.3 ± 0.2^aB^
Harvest	320.8 ± 25.0^aC^	308.33 ± 21.5^aC^	19.8 ± 1.2^aB^	18.0 ± 1.4^aB^	16.4 ± 0.7^aA^	15.1 ± 0.7^bA^	7.88 ± 0.2^aB^	7.91 ± 0.2^aB^	6.1 ± 0.2^aC^	5.8 ± 0.1^aA^
Mean of soybean	327.8^a^	306.9^a^	18.6^a^	17.0^a^	16.7^a^	14.9^b^	8.01^a^	8.01^a^	6.4^a^	6.1^a^
**Wheat**										
60 DAS	325.0 ± 28.7^aB^	312.5 ± 16.0^aB^	22.2 ± 1.2^aB^	20.4 ± 1.4^aB^	16.9 ± 0.4^aA^	16.4 ± 0.3^aA^	8.12 ± 0.5^aA^	7.83 ± 0.1^bC^	5.6 ± 0.1^aC^	5.1 ± 0.1^aA^
Harvest	250.0 ± 13.6^aA^	237.5 ± 25.0^aA^	17.4 ± 1.2^aA^	16.2 ± 1.2^aA^	15.6 ± 0.4^aB^	15.0 ± 0.5^aB^	8.07 ± 0.1^aA^	8.04 ± 0.2^aA^	5.0 ± 0.1^aA^	4.8 ± 0.1^aC^
Mean of wheat	287.5^a^	275^a^	19.8^a^	18.3^a^	16.25^a^	15.7^a^	8.1^a^	7.9^a^	5.3^a^	5.0^a^


*Data are mean values of four replicates; values followed by the same small letters are not significantly different among organic and conventional practices at *P* = 0.05 (horizontal comparison). Similarly values followed by the same capital letters are not significantly different between different periods of sampling (vertical comparison). Values under ANOVA are probabilities (*P*-values) of the source of variations.*

Soil pH did not differ significantly between organic and conventional management practices in soybean at any stage, however, a significant decline in both the management systems was observed at harvest of soybean (**Table 2**). The available phosphorus content was highest at R2 stage of soybean, irrespective of the production system. Though the availability of P tended to be slightly higher under organic management at different sampling points, the differences were not statistically significant (**Table 2**). Seed yield of soybean was statistically similar under organic (1902 kg ha^-1^) and conventional management (1848 kg ha^-1^). Similarly, soybean straw yield was also comparable in organic (1756 kg ha^-1^) and conventional system (1723 kg ha^-1^). However, in case of wheat, conventional system produced significantly higher seed and straw yield than organic.

**FIGURE 2:**
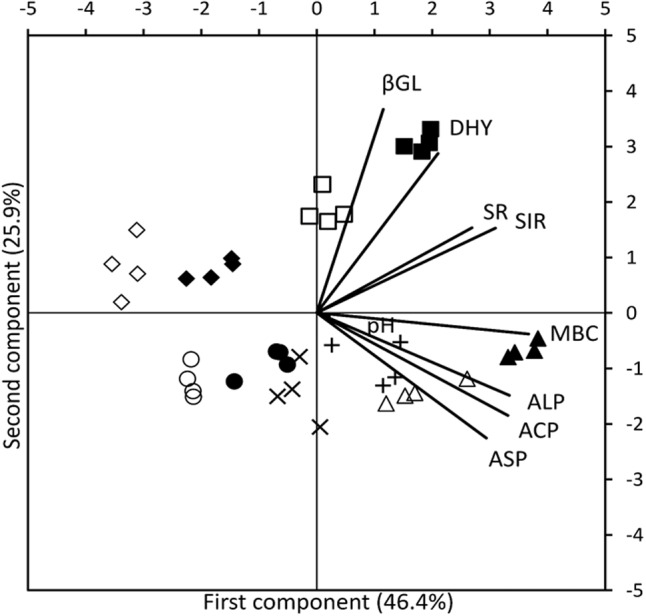
Bi-plot for the principal component analysis of organic and conventional soybean and wheat management systems at five different times of sampling: Before sowing of Soybean (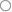 CONV, ●ORG), 50 DAS (△ CONV, 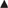 ORG), before sowing of wheat (× CONV, + ORG), 60 DAS (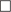 CONV, 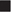 ORG), and after wheat harvest (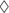 CONV, 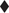 ORG). Soil pH (pH), microbial biomass carbon (MBC), acid phosphatase (ACP), alkaline phosphatase (ALP), arylsulphatase (ASP), β-glucosidase (βGL), dehydrogenase (DHA), microbial soil respiration (SR), and substrate induced respiration (SIR).

The results of PCA analysis provided a comprehensive picture of parameters that work synergistically in each management system. In the bi-plot (**Figure 2**), length of the vector corresponding to a particular soil parameter demonstrates the extent of relative contribution of that parameter. Whereas, the proximity of a vector to a symbol cluster indicates the association of that biological parameter to the particular farming system and sampling time represented by that symbol cluster. Cumulative variability of 84.2% was captured by first three principal components (PC) (**Table 3**). Distinguished presence of farming systems’ clustered replicates in different quadrates indicated the extent of activities of variables at different sampling times (**Figure 2**). Dissociation between systems and variables at soybean sowing, wheat harvest and wheat sowing (only conventional) clearly came out in PCA from the presence of respective points in 2nd and 3rd quadrate, which are aloof from vectors of variables. In organic systems, the main active variable selected by PCA was the MBC at R2 stage of soybean and DHA was the main active variable at panicle initiation stage of wheat (**Figure 2**). No such association of a particular variable at active crop growth stages of soybean and wheat was found in conventional system. The first PC explained 46.6% of variability with major contribution of MBC, ACP, and ALP. In 2nd component major contribution comes from βGL and DHA which explained variability of 25.9%. Soil pH was the only major contributor for the PC3 and explained the variability of 11.9%.

**Table 3:** Eigenvectors corresponding to principal components including eigenvalues and cumulative proportion variance of measured variables.

Variables	PC		
	
	1	2	3
pH	0.03	-0.01	0.95
MBC	0.45	-0.06	-0.01
ACP	0.40	-0.30	-0.07
ALP	0.41	-0.24	0.05
ASP	0.36	-0.37	0.11
βGL	0.14	0.60	0.07
DHA	0.26	0.47	0.14
SR	0.33	0.25	-0.03
SIR	0.38	0.25	-0.23
Eigenvalue	4.18	2.33	1.07
Cumulative proportion	46.4	72.3	84.2


## Discussion

The overall hypothesis of this study was supported by finding of higher biological activity in organic systems that resulted in attaining P availability equivalent to conventional systems. Soil microbiological parameters such as DHA, βGL, ACP, ALP, SIR, SR, and MBC were in general higher in soil of plots under 7 years of organic management compared to those under conventional systems, particularly at key growth stages of both soybean and wheat crops. Soil enzymes have been suggested as potential indicators of soil quality because of their ease of measurements, relationship to belowground microbiological processes and rapid response to changes in agricultural management (Dick et al., 1996; Dick, 1997; Jimenez et al., 2002). Measurement of soil enzyme activities also provides an integrative response to changes in soil chemical, physical and biological characteristics under different management induced perturbations and is used to monitor the effects of different agricultural management strategies on long-term productivity (Doran and Parkin, 1994; Ndiaye et al., 2000; Acosta-Martinez et al., 2007). These measurements also provide credible information on the key reactions that participate in the rate limiting steps in the decomposition of soil organic matter and nutrient transformation in soils and are thus of high relevance in understanding P availability under different management systems. The soil enzyme activities measured in this study increased from sowing to R2 stage in soybean and to panicle initiation stage in wheat crop and again declined toward harvest. This increase in soil enzyme activities during active crop growth stages can be ascribed to increased rhizo-deposition (Gregory, 2006; Mandal et al., 2007; Nayak et al., 2007; Masto et al., 2013; Tamilselvi et al., 2015). The higher enzyme activity in organic agricultural system can also be attributed to enhanced nutrient availability from added organic inputs, increased root exudation owing to improved crop growth and conducive environment for microbial proliferation (Burns et al., 2013; Tamilselvi et al., 2015). PCA results showed that DHA was main contributing factor in organic systems at panicle initiation stage of wheat. Dehydrogenase is an oxidoreductase enzyme that is present only in viable cells and measurement of DHA provides an index of endogenous soil microbial activity as its assay involves no addition of substrate that preferentially stimulates any particular group of soil microorganisms (Biederbeck et al., 2005). For this reason, DHA assay has been used as a potential soil quality indicator to discriminate changes under different agricultural management systems (Kandeler et al., 1999; Aseri and Tarafdar, 2006; Aparna et al., 2014).

Similarly, βGL is involved in decomposition of cellulose compounds and is synthesized by soil microorganisms in the presence of suitable substrates. Therefore, it has been used as sensitive indicator of microbially mediated soil processes (Sinsabaugh, 1994; Lagomarsino et al., 2009; Stott et al., 2010). Phosphatase activity in the soils may originate either from plant roots or from microorganisms such as fungi and bacteria (Tarafdar and Chhonkar, 1979; Tarafdar et al., 1988; Dinkelaker and Marschner, 1992) and changes in its activity could indicate changes in the quantity and quality of soil phosphoryl substrates (Rao and Tarafdar, 1992). Apart from creating conducive environment for increased biological activity, organic amendments are rich in microbial biomass and may also contain intra- and extracellular enzymes that stimulate microbial activity in soil (Liang et al., 2005; Tejada et al., 2006). Our findings are consistent with earlier studies that showed an increase in enzyme activities with the application of organic amendments (Marinari et al., 2006; Tejada et al., 2006; Aparna et al., 2014).

Generally, organic inputs increase C and energy availability to microorganisms, thereby stimulating indigenous soil microbial biomass and activity, especially in C-depleted agricultural soils. In a long-term study conducted under temperate environmental conditions, Fließbach and Mäder (2000), found 45–64% higher microbial biomass in bio-dynamic farming systems than conventional systems after18 years of respective crop management. In contrast, our results show only an average increase of about 6% MBC under organic management after 7 years of experimentation, while the differences are non-significant at individual sampling points. This indicates that due to higher turnover rates under tropical environments (as in our study), 7 years is probably not a long enough period to see clearly distinguishable differences in MBC. Moreover, owing to the concept of good agricultural practices, conventionally managed plots in this field trial also receives four tons of FYM every alternate year (Forster et al., 2013), which contributes to MBC in conventional plots and hence might have acted as a confounding factor minimizing differences among productions systems. Nevertheless, PCA results showed that MBC was the main factor contributing to biological activities at R2 stage of soybean in organic systems. Soil microbial respiration rate gives an indication of microbiological activity in the soil and is influenced by carbon availability to microorganisms in the soil environment. We found higher rates of microbial respiration in organically amended soils, which could be attributed to greater labile fractions of organic matter in the added organic manures (Tu et al., 2006; Chinnadurai et al., 2014; Tamilselvi et al., 2015; Hernández et al., 2016). Similarly, SIR, another soil quality indicator that provides us information on the metabolic and physiological state of soil microorganisms (Anderson, 1994), tended to be higher under organic management (Chinnadurai et al., 2014; Tamilselvi et al., 2015). Moreover, both SR and SIR were found to be significantly higher at active crop growth stages that could be attributed to increased rhizo-deposition which is conducive for microbial proliferation (Mandal et al., 2007; Li et al., 2012; Masto et al., 2013; Tamilselvi et al., 2015).

Soil pH is considered an important factor influencing P availability in soils and it could play a crucial role in alkaline soils of our experimental site. However, in this study the differences in pH among organic and conventional systems on an average were not significant enough to exert a major influence *per se*. The most interesting observation in this regard was the dip in pH at the harvest of soybean, which recovered in organic systems (8.12) in the subsequent sampling (panicle initiation stage of wheat) but not in conventional (7.83). The reduction in pH at the harvest of soybean is plausible as the leguminous plants are known to reduce soil pH (Yan et al., 1996; Opala et al., 2012). However, the observed differences in pH at panicle initiation stage of wheat seem strongly influenced by management practices. The organic systems received a basal dose of FYM based compost at the planting of wheat, which seems to have contributed to quick recovery in pH (Whalen et al., 2000). Whereas, conventional systems received a basal dose of mineral P and K fertilizers (SSP and MOP) at sowing of wheat and two split doses of N (Urea) at 19 and 43 DAS, which might have resulted in a lower pH. Use of acidifying inorganic mineral fertilizers over considerably longer periods is known to result in a decline in soil pH (Birkhofer et al., 2008), which could in turn affect aggregate stability and loss of organic matter (Mäder et al., 2002; Mikha and Rice, 2004). Inputs of organic manures applied every alternate year to conventional plots in this study might be an important contributing factor in slowing down the acidification of soil over longer term.

On an average, P availability in the soil under organic management tended to be higher (5.9 μg g^-1^) than that under conventional management (5.6 ± 0.1 μg g^-1^). However, at any particular sampling time, differences in P availability among the two management systems were not statistically significant. It is noteworthy that despite the application of mineral P in conventional plots at sowing of wheat, the availability of P tended to be slightly lower than that under organic management. The values of P availability at panicle initiation stage of wheat under organic (5.6 μg g^-1^) and conventional (5.1 μg g^-1^) management indicate that most of the P applied to conventional plots in the form of mineral fertilizer was either utilized by the crop or fixed by the soil. Since yield of wheat was considerably higher under conventional management, it is plausible that the P applied at sowing was taken up by the crop by panicle initiation stage. Comparing the P availability among two management systems at all the five sampling times, we can conclude that P availability was not a limiting factor for organic at any of these time points. However, utilization of available P by crop plants depends on several factors and N availability could be one of them (Riar and Coventry, 2012). Since soybean can symbiotically assimilate atmospheric nitrogen, probably it had relatively higher capability of utilizing available P compared to wheat. Therefore, soybean yield under organic management was equivalent to conventional management, which was not the case of wheat. Further investigations would be needed to identify the factors responsible for yield difference in wheat, however, yield is a complex trait influenced by multiple factors discussion of which is beyond the scope of this study.

From the findings of this study, we conclude that owing to higher biological activity, organically managed agricultural soils could attain equivalent or higher P availability than conventionally managed soils receiving regular inputs of mineral P fertilizers. These results are of particular relevance to alkaline vertisols, wherein sorption and precipitation are important influencing factors in determining the availability of P. These findings also carry a high global applicability, for instance, P-fixing soils are widely prevalent in Africa, where P-inputs through mineral fertilizers are ineffective. Organic management over a considerable time period could support in building up fertility and enhancing P availability in these soils. Moreover, it also offers a suitable alternative to resource poor small holder farmers of developing countries who cannot afford the expensive mineral fertilizers.

## Author Contributions

NB, GB, and SI conceived the project. NB conducted the field study and lab work. AkR, MS, and SS provided scientific support for the lab work. NB, AkR, MS, and AmR analyzed the data. NB, AkR, AmR, and GB wrote the manuscript and all authors revised the manuscript.

## Conflict of Interest Statement

The authors declare that the research was conducted in the absence of any commercial or financial relationships that could be construed as a potential conflict of interest.

## References

[B1] Acosta-Martinez V. Cruz L. Ramirez D. S. Alegria L. P. (2007). Enzyme activities as affected by soil properties and land use in a tropical watershed. *Appl. Soil Ecol.* 35 35–45. 10.1016/j.apsoil.2006.05.012

[B2] Alam M. M. Ladha J. K. (2004). Optimizing phosphorus fertilization in an intensive vegetable–rice cropping system. *Biol. Fertil. Soils* 40 277–283. 10.1007/s00374-004-0778-7

[B3] Anderson J. P. E. Domsch K. H. (1978). A physiological method for the quantitative measurement of microbial biomass in soils. *Soil Biol. Biochem.* 75 43–48. 10.1016/0038-0717(78)90099-8

[B4] Anderson T. H. (1994). “Physiological analysis of microbial communities in soil: application and limitations,” in *Beyond the Biomass.* eds Ritz K. Dighton J. Giller K. E. (Exeter: Wiley-Sayce), 67–76.

[B5] Anderson T. H. Domsch K. H. (1990). Application of eco-physiological quitients (qCO2 and qD) on microbial biomasses from soils of different cropping histories. *Soil Biol. Biochem.* 25 393–395. 10.1016/0038-0717(93)90140-7

[B6] Aparna K. Pasha M. A. Rao D. L. N. Krishnaraj P. U. (2014). Organic amendments as ecosystem engineers: microbial, biochemical and genomic evidence of soil health improvement in a tropical arid zone field site. *Ecol. Eng.* 71 268–277. 10.1016/j.ecoleng.2014.07.016

[B7] Aseri G. K. Tarafdar J. C. (2006). Fluoresciencediacetate: a potential biological indicator for arid soils. *Arid Land Res. Manag.* 20 87–99. 10.1007/s00248-011-9867-y

[B8] Aulakh M. S. Kabba B. S. Baddesha H. S. Bahl G. S. Gill M. P. S. (2003). Crop yields and phosphorus fertilizer transformations aft er 25 years of applications to a subtropical soil under groundnut based cropping systems. *Field. Crops Res.* 83 283–296. 10.1016/S0378-4290(03)00078-9

[B9] Bai Z. Li H. Yang X. Zhou B. Shi X. Wang B. (2013). The critical soil P levels for crop yield, soil fertility and environmental safety in different soil types. *Plant Soil* 372 27–37. 10.1007/s11104-013-1696-y

[B10] Baruah T. C. Barthakur H. P. (1999). *A Text Book of Soil Analysis.* New Delhi: Vikas Publishing House Pvt. Ltd.

[B11] Basamba T. A. Barrios E. Amezquita E. I. Rao M. Singh B. R. (2006). Tillage effects on maize yield in a Colombian savanna Oxisol: soil organic matter and P fractions. *Soil Tillage Res.* 91 131–142. 10.1016/j.still.2005.11.010

[B12] Biederbeck V. O. Zetner R. P. Campbell C. A. (2005). Soil microbial populations and activities as influenced by legume green fallow in a semi-arid climate. *Soil Biol. Biochem.* 37 1775–1784. 10.1016/j.soilbio.2005.02.011

[B13] Birkhofer K. Bezemer T. M. Bloem J. Bonkowski M. Christensen S. Dubois D. (2008). Long-term organic farming fosters below and aboveground biota: implications for soil quality, biological control and productivity. *Soil Biol. Biochem.* 40 2297–2308. 10.1016/j.soilbio.2008.05.007

[B14] Bolan N. S. Naidu R. Mahimairaja S. Baskaran S. (1994). Influence of low molecular- weight organic acids on the solubilization of phosphates. *Biol. Fertil Soils* 18 311–319. 10.1590/S1517-838246246220131102

[B15] Brady N. C. Weil R. R. (2008). *The Nature and Properties of Soils*, 4th Edn Upper Saddle River, NJ: Prentice Hall.

[B16] Burns R. G. DeForest J. L. Marxsen J. Sinsabaugh R. L. Stromberger M. E. Wallemstein M. D. (2013). Soil enzymes in a changing environment: current knowledge and future directions. *Soil Biol. Biochem.* 32 1547–1559. 10.1016/j.soilbio.2012.11.009

[B17] Chinnadurai C. Gopalaswamy G. Balachandar D. (2014). Impact of long-term organic and inorganic nutrient managements on the biological properties and eubacterial community diversity of the Indian semi-arid Alfisol. *Arch. Agron. Soil Sci.* 60 531–548. 10.1080/03650340.2013.803072

[B18] Ciampitti I. A. García F. O. Picone L. I. Rubi G. (2011). Phosphorus budget and soil extractable dynamics in field crop rotations in mollisols. *Soil Sci. Soc. Am. J.* 75 131–142. 10.2136/sssaj2009.0345

[B19] Condron L. M. Turner B. L. Cade-Menum B. J. (2005). “Chemistry and dynamics of soil organic phosphorus,” in *Phosphorus: Agriculture and the Environment*, eds Sims J. T. Sharpley A. N. (Madison, WI: American Society of Agronomy), 87–121.

[B20] Cordell D. Drangert J. O. White S. (2009). The story of phosphorus: global food security and food for thought. *Glob. Environ. Change* 19 292–305. 10.1016/j.gloenvcha.2008.10.009

[B21] Damodar Reddy D. Subba Rao A. Sammi Reddy K. Takkar P. N. (1999). Yield sustainability and phosphorus utilization in soybean-wheat system on vertisols in response to integrated use of manure and fertilizer phosphorus. *Field Crops Res.* 62 181–190. 10.1016/S0378-4290(99)00019-2

[B22] Dick R. P. (1997). “Soil enzyme activities as integrative indicators of soil health,” in *Biological Indicators of Soil Health*, eds Pankhurst C. E. Doube B. M. Gupta V. V. S. R. (Wellingford: CABI), 121–156.

[B23] Dick R. P. Breakwell D. Turco R. (1996). “Soil enzyme activities and biodiversity measurements as integrating biological indicators,” in *Handbook of Methods for Soil Assessment of Soil Quality*, eds Doran J. W. Jones A. J. (Madison, WI: Soil Science Society of America), 247–272.

[B24] Dick R. P. Rasmussen P. E. Kerle E. A. (1988). Influence of long-term residue management on soil enzyme activities in relation to soil chemical properties of a wheat-fallow system. *Biol. Fertil. Soils* 6 159–164. 10.1007/BF00257667

[B25] Dinkelaker B. Marschner H. (1992). In vivo demonstration of acid phosphatase activity in the rhizosphere of soil grown plants. *Plant Soil* 144 199–205. 10.1007/BF00012876

[B26] Doran J. W. Parkin T. B. (1994). “Defining and assessing soil quality,” in *Defining Soil Quality for A Sustainable Environment*, eds Doran J. W. Coleman D. C. Bezdicek D. F. Stewart B. A. (Madison, WI: Soil Science Society of America Special Publication), 3–21.

[B27] Fließbach A. Mäder P. (2000). Microbial biomass and size density fractions differ between soils of organic and conventional agricultural systems. *Soil Biol. Biochem.* 32 757–768. 10.1016/S0038-0717(99)00197-2

[B28] Forster D. Andres C. Verma R. Zundel C. Messmer M. M. Mäder P. (2013). Yield and economic performance of organic and conventional cotton-based farming systems - Results from a field trial in India. *PLoS ONE* 8:e81039 10.1371/journal.pone.0081039 PMC3852008 24324659

[B29] George T. S. Richardson A. E. Simpson R. J. (2005). Behaviour of plant-derived 621 extracellular phytase upon addition to soil. *Soil Biol. Biochem.* 37 977–988. 10.1016/j.soilbio.2004.10.016

[B30] Gichangi E. M. Mnkeni P. N. S. Brookes P. C. (2009). Effects of goat manure and inorganic phosphate addition on soil inorganic and microbial biomass phosphorus fractions under laboratory incubation conditions. *Soil Sci. Plant Nutr.* 55 764–771. 10.1111/j.1747-0765.2009.00415.x

[B31] Goupy J. Creighton L. (2007). *Introduction to Design of Experiments with JMP Examples*, 3rd Edn Cary, NC: SAS Institute Inc.

[B32] Gregory P. J. (2006). *Plant Roots: Growth, Activity and Interactions with Soils.* Oxford: Black-well publishing Ltd., 216 10.1002/9780470995563

[B33] Guan G. Tu S. Li H. Yang J. Zhang J. Wen S. (2012). Phosphorus fertilization modes affect crop yield, nutrient uptake, and soil biological properties in the rice–wheat cropping system. *Soil Sci. Soc. Am. J.* 77 166–172. 10.2136/sssaj2011.0324

[B34] Harvey P. R. Warren R. A. Wakelin S. (2009). Potential to improve root access to phosphorus: the role of non-symbiotic microbial inoculants in the rhizosphere. *Crop Pasture Sci.* 60 144–151. 10.1071/CP08084

[B35] Hernández T. Chocano C. Moreno J. L. Garcia C. (2016). Use of compost as an alternative to conventional inorganic fertilizer in intensive lettuce (*Lactuca sativa* L.) crops- Effects on soil and plant. *Soil Tillage Res.* 160 14–22. 10.1016/j.still.2016.02.005

[B36] Hinsinger P. (2001). Bioavailability of soil inorganic P in the rhizosphere as affected by root-induced chemical changes: a review. *Plant Soil* 237 173–195. 10.1023/A:1013351617532

[B37] IFOAM (2006). *The IFOAM Norms for Organic Production and Processing, Version 2005.* Bonn: Die Deutsche Bibliothek.

[B38] Iyamuremye F. Dick R. P. (1996). Organic amendments and phosphorus sorption by soils. *Adv. Agron.* 56 139–185. 10.1016/S0065-2113(08)60181-9

[B39] Jimenez M. P. Horra A. M. Pruzzo L. Palma R. M. (2002). Soil quality: a new index based on microbiological and biochemical parameter. *Biol. Fertil. Soils* 35 302–306. 10.1007/s00374-002-0450-z

[B40] Joergensen R. G. Mueller T. (1996). The fumigation-extraction method to estimate soil microbial biomass: calibration of the kEC value. *Soil Biol. Biochem.* 28 33–37. 10.1016/0038-0717(95)00101-8

[B41] Johnston A. E. Poulton P. R. Fixen P. E. Curtin D. (2014). Phosphorus: its efficient use in agriculture. *Adv. Agron.* 123 177–228. 10.1016/B978-0-12-420225-2.00005-4

[B42] Kandeler E. Stemmer M. Klimanek E. M. (1999). Response of soil microbial biomass, urease and xylanase within particle fraction to long-term management. *Soil Biol. Biochem.* 31 261–273. 10.1016/S0038-0717(98)00115-1

[B43] Khan K. S. Joergensen R. G. (2009). Changes in microbial biomass and P fractions in biogenic household waste compost amended with inorganic P fertilizers. *Biores. Technol.* 100 303–309. 10.1016/j.biortech.2008.06.002 18632264

[B44] Klein D. A. Loh T. C. Goulding R. L. (1971). A rapid procedure to evaluate dehydrogenase activity of soils low in organic matter. *Soil Biol. Biochem.* 3 385–387. 10.1016/0038-0717(71)90049-6

[B45] Kong L. Wang Y. B. Zhao L. N. Chen Z. H. (2009). Enzyme and root activities in surface-flow constructed wetlands. *Chemosphere* 76 601–608. 10.1016/j.chemosphere.2009.04.056 19497608

[B46] Kumar A. Suri V. K. Choudhary A. K. (2014). Influence of inorganic phosphorus, VAM fungi, and irrigation regimes on crop productivity and phosphorus transformations in okra (*Abelmoschus esculentus* L.)–Pea (*Pisum sativum* L.) cropping system in an acid alfisol. *Commun. Soil Sci. Plant Anal.* 45 953–967. 10.1080/00103624.2013.874025

[B47] Lagomarsino A. Moscatellil M. C. Di Tizio A. Mancinelli R. Greglo S. Marinaril S. (2009). Soil biochemical indicators as a tool to assess the short-term impact of agricultural management on changes in organic C in a Mediterranean environment. *Ecol. Indic.* 9 518–527. 10.1016/j.ecolind.2008.07.003

[B48] Li X. H. Han X. Z. Li H. B. Song C. Yan J. Liang Y. (2012). Soil chemical and biological properties affected by 21-year application of composted manure with chemical fertilisers in a Chinese mollisol. *Can. J. Soil Sci.* 92 419–428. 10.4141/cjss2010-046

[B49] Liang Y. Nikolic M. Peng Y. Chen W. Jiang Y. (2005). Organic manure stimulates biological activity and barley growth in soil subject to secondary salinization. *Soil Biol. Biochem.* 37 1185–1195. 10.1016/j.soilbio.2004.11.017

[B50] Lynch J. P. Brown K. M. (2008). “Root strategies for phosphorus acquisition,” in *The Ecophysiology of Plant-Phosphorus Interactions*. eds White P. J. Hammond J. P. (Dordrecht: Springer), 83–116. 10.1007/978-1-4020-8435-5_5

[B51] Mäder P. Fliessbach A. Dubois D. Gunst L. Fried P. Niggli U. (2002). Soil fertility and 529 biodiversity in organic farming. *Science* 296 1694–1697. 10.1126/science.1071148 12040197

[B52] Malik M. A. Khan K. S. Marshchner P. Ali S. (2013). Organic amendments differ in their effect on microbial biomass and activity and on P pools in alkaline soils. *Biol. Fertil. Soils* 49 415–425. 10.1007/s00374-012-0738-6

[B53] Malik M. A. Marschner P. Khan K. S. (2012). Addition of organic and inorganic P sources to soil- effects on P pools and microorganisms. *Soil Biol. Biochem.* 49 106–113. 10.1016/j.soilbio.2012.02.013

[B54] Mandal A. Patra A. K. Singh D. Swarup A. Masto R. E. (2007). Effect of long-term application of manure and fertilizer on biological and biochemical activities in soil during criop development stages. *Bioresour. Technol.* 98 3585–3592. 10.1016/j.biortech.2006.11.027 17207997

[B55] Marinari S. Mancinelli R. Campiglia E. Grego S. (2006). Chemical and biological indicators of soil quality in organic and conventional farming systems in Central Italy. *Ecol. Indic.* 6 701–711. 10.1016/j.ecolind.2005.08.029

[B56] Masto R. E. Ansari M. A. George J. Selvi V. A. Ram L. C. (2013). Co-application of biochar and lignite fly ash on soil nutrients and biological parameters at different crop growth stages of *Zea mays*. *Ecol. Eng.* 58 314–322. 10.1016/j.ecoleng.2013.07.011

[B57] Mikha M. M. Rice C. W. (2004). Tillage and manure effect on soil and aggregate associated carbon and nitrogen. *Soil Sci. Soc. Am. J.* 68 809–816. 10.2136/sssaj2004.8090

[B58] Nannipieri P. (1994). “The potential use of soil enzymes as indicators of productivity, sustainability and pollution,” in *Soil Biota: Management in Sustainable Farming Systems*, eds Pankhurst C. E. Doube B. M. Gupta V. V. S. R. Grace P. R. (East Melbourne, VIC: CSIRO), 238–244.

[B59] Nayak D. R. Babu Y. J. Adhya T. K. (2007). Long-term application of compost influences microbial biomass and enzyme activities in a tropical Aeric Endoaquept planted to rice under flooded condition. *Soil Biol. Biochem.* 39 1897–1906. 10.1016/j.soilbio.2007.02.003

[B60] Ndiaye E. L. Sandeno J. M. Mcgrath D. Dick R. P. (2000). Integrative biological indicators for detecting changes in soil quality. *Am. J. Alternative Agric.* 15 26–36. 10.1017/S0889189300008432

[B61] Olsen S. R. Cole C. V. Watanabe F. S. Dean L. A. (1954). Estimation of available P in soils by extraction with sodium bicarbonates. *Circular* 939 1–19.

[B62] Opala P. A. Okalebo J. R. Othieno C. O. (2012). Effects of organic and inorganic materials on soil acidity and phosphorus availability in a soil incubation study. *ISRN Agron.* 2012:597216 10.5402/2012/597216

[B63] Ramesh A. Sharma S. K. Joshi O. P. Khan I. R. (2011). Phytase, phosphatase activity and P-nutrition of soybean as influenced by inoculation of *Bacillus*. *Indian J. Microbiol.* 51 94–99. 10.1007/s12088-011-0104-7 22282635 PMC3209857

[B64] Ramesh A. Sharma S. K. Yadav N. P. Joshi O. P. (2014). Phosphorus mobilization from native soil P-pool upon inoculation with phytate-mineralizing and phosphate-solubilizing *Bacillus aryabhattai* isolates for improved P-acquisition and growth of soybean and wheat crops in microcosm conditions. *Agric. Res.* 3 118–127. 10.1007/s40003-014-0105-y

[B65] Rao A. V. Tarafdar J. C. (1992). Seasonal changes in available phosphorus and different enzyme activities in arid soils. *Ann. Arid Zone.* 31 185–189.

[B66] Riar A. Coventry D. (2012). “Nitrogen use as a component of sustainable crop systems,” in *Agricultural Sustainability: Progress and Prospects in Crop Research*, eds Bhullar G. S. Bhullar N. K. (London: Academic Press), 63–76.

[B67] Richardson A. Lynch J. Ryan P. Delhaize E. Smith F. Smith S. (2011). Plant and microbial strategies to improve the phosphorus efficiency of agriculture. *Plant Soil* 349 121–156. 10.2527/jas.53804

[B68] Richardson A. E. (1994). “Soil microorganisms and phosphorus availability,” in *Soil Biota Management in Sustainable Farming Systems*, eds Pankhurst C. E. Doubeand B. M. Gupta V. V. S. (Melbourne, VIC: CSIRO Publishing), 50–62.

[B69] Sánchez P. A. (2010). Tripling crop yields in tropical Africa. *Nat. Geosci.* 3 299–300. 10.1098/rstb.2015.0316

[B70] Shen J. Yuan L. Zhang J. Li H. Bai Z. Chen X. (2011). Phosphorus dynamics: from soil to plant. *Plant Physiol.* 156 997–1005. 10.1104/pp.111.175232 21571668 PMC3135930

[B71] Shi Y. Ziadi N. Messiga A. J. Lalande R. Hu Z. (2013). Changes in soil phosphorus fractions for a long-term corn-soybean rotation with tillage and phosphorus fertilization. *Soil Sci. Soc. Am. J.* 77 1402–1412. 10.2136/sssaj2012.0427

[B72] Singh M. Reddy K. S. Singh V. P. Rupa T. R. (2007). Phosphorus availability to rice (*Oriza sativa* L.)-wheat (*Triticum estivum* L.) in a Vertisol after eight years of inorganic and organic fertilizer additions. *Bioresour. Technol.* 98 1474–1481. 10.1016/j.biortech.2006.02.045 17067794

[B73] Sinsabaugh R. L. Klug M. J. Collins H. P. Yeager P. E. Petersen S. O. (1999). “Characterizing soil microbial communities,” in *Standard Soil Methods for Long Term Ecological Research*, eds Robertson G. P. Bledsoe C. S. Coleman D. C. Sollins P. (New York: Oxford University Press), 318–348.

[B74] Sinsabaugh R. S. (1994). Enzymic analysis of microbial pattern and process. *Biol. Fertil. Soils* 17 69–74. 10.1007/BF00418675

[B75] Stott D. E. Andrews S. S. Liebig M. A. Wienhold B. J. Karlen D. L. (2010). Evaluation of β-glucosidase activity as a soil quality indicator for the soil management assessment framework. *Soil Sci. Soc. Am. J.* 74 107–119. 10.2136/sssaj2009.0029

[B76] Syers J. K. Johnston A. E. Curtin D. (2008). *Efficiency of Soil and Fertilizer Phosphorus Use: Reconciling Changing Concepts of Soil Phosphorus Behaviour with Agronomic Information.* Rome: FAO.

[B77] Tabatabai M. A. (1994). “Soil enzymes,” in *Methods of Soil Analysis: Microbiological and Biochemical Properties*, eds Weaver R. W. Angle J. S. Bottomley P. S. (Madison, WI: Soil Science Society of America), 775–833.

[B78] Tabatabai M. A. Bremner J. M. (1969). Use of p-nitrophenylphosphate for assay of soil phosphatase activity. *Soil Biol. Biochem.* 1 301–307. 10.1186/1756-0500-7-221

[B79] Tamilselvi S. M. Chinnadurai C. Hamuruga K. Arulmozhiselvan K. Balachandran D. (2015). Effect of long-term nutrient management on biological and biochemical properties of semi-arid tropical Alfisol during maize crop development stages. *Ecol. Indic.* 48 76–87. 10.1016/j.ecolind.2014.08.001

[B80] Tang J. Leung A. Leung C. Lim B. L. (2006). Hydrolysis of precipitated phytate by three distinct families of phytases. *Soil Biol. Biochem.* 38 1316–1324. 10.1016/j.soilbio.2005.08.021

[B81] Tarafdar J. C. Chhonkar P. K. (1979). Phosphatase production by microorganisms isolated from diverse types of soils. *Zentralbl. Bacteriol.* 134 119–124. 10.1016/S0323-6056(79)80037-3 224622

[B82] Tarafdar J. C. Claassen N. (1988). Organic phosphorus compounds as a phosphorus source for higher plants through the activity of phosphatases produced by plant roots and microorganisms. *Biol. Fertil. Soils* 5 308–312. 10.1007/BF00262137

[B83] Tarafdar J. C. Rao A. V. Bala K. (1988). Production of phosphatates by fungi isolated from desert soils. *Folia Microbiol.* 33 453–457. 10.1007/BF02925770

[B84] Tejada M. Hernandez M. T. Garcia C. (2006). Application of two organic amendments on soil restoration: effects on soil biological properties. *J. Environ. Qual.* 35 1010–1017. 10.2134/jeq2005.0460 16738385

[B85] Tu C. Louws F. J. Creamer N. G. Mueller J. P. Brownie C. Fager K. (2006). Responses of soil microbial biomass and N availability to transition strategies from conventional to organic farming systems. *Agric. Ecosyst. Environ.* 113 206–215. 10.1016/j.agee.2005.09.013

[B86] Turner B. J. McKelvie I. D. Haygarth P. M. (2002). Characterisation of water extractable soil organic phosphorus by phosphatase hydrolysis. *Soil Biol. Biochem.* 34 27–35. 10.1016/S0038-0717(01)00144-4

[B87] Turner B. L. Newman S. Cheesman A. W. Reddy K. R. (2007). Sample pretreatment and phosphorus speciation in wetland soils. *Soil Sci. Soc. Am. J.* 71 1538–1546. 10.2136/sssaj2007.0017

[B88] Vance E. D. Brookes P. C. Jenkinson D. S. (1987). An extraction method for measuring soil microbial biomass. *Soil Biol. Biochem.* 19 703–707. 10.1016/0038-0717(87)90052-6

[B89] Watanabe F. S. Olsen S. R. (1965). Test of an ascorbic acid method for determining phosphorus in water and NaHCO3 extracts from soil. *Soil Sci. Soc. Am. J. Proc.* 29 677–678. 10.2136/sssaj1965.03615995002900060025x

[B90] Whalen J. K. Chang C. Clayton G. W. Carefoot J. P. (2000). Cattle manure amendments can increase the pH of acid soils. *Soil Sci. Soc. Am. J.* 64 962–966. 10.2136/sssaj2000.643962x

[B91] Yan F. Schubert S. Mengel K. (1996). Soil pH changes during legume growth and application of plant material. *Biol. Fertil. Soils* 23 236–242. 10.1007/BF00335950

[B92] Zhang F. Shen J. Zhang J. Zuo Y. Li L. Chen X. (2010). Chapter one-rhizosphere processes and management for improving nutrient use efficiency and crop productivity: implications for China. *Adv. Agron.* 107 1–32. 10.1016/S0065-2113(10)07001-X

